# Activation of skeletal muscle is controlled by a dual-filament mechano-sensing mechanism

**DOI:** 10.1073/pnas.2302837120

**Published:** 2023-05-22

**Authors:** Elisabetta Brunello, Lorenzo Marcucci, Malcolm Irving, Luca Fusi

**Affiliations:** ^a^Randall Centre for Cell and Molecular Biophysics, School of Basic and Medical Biosciences and British Heart Foundation Centre of Research Excellence, King’s College London, London SE1 1UL, United Kingdom; ^b^Department of Biomedical Sciences, University of Padova, Padova 35131, Italy; ^c^RIKEN Centre for Biosystems Dynamics Research, Suita 565-0874, Japan; ^d^Centre for Human and Applied Physiological Sciences, School of Basic and Medical Biosciences, King’s College London, London SE1 1UL, United Kingdom

**Keywords:** skeletal muscle, troponin, myosin, muscle regulation

## Abstract

Contraction of skeletal muscle is triggered by a transient rise in intracellular calcium concentration leading to a structural change in the actin-containing thin filaments that allows binding of myosin motors from the thick filaments. Most myosin motors are unavailable for actin binding in resting muscle because they are folded back against the thick filament backbone. Release of the folded motors is triggered by thick filament stress, implying a positive feedback loop in the thick filaments. However, it was unclear how thin and thick filament activation mechanisms are coordinated, partly because most previous studies of the thin filament regulation were conducted at low temperatures where the thick filament mechanisms are inhibited. Here, we use probes on both troponin in the thin filaments and myosin in the thick filaments to monitor the activation states of both filaments in near-physiological conditions. We characterize those activation states both in the steady state, using conventional titrations with calcium buffers, and during activation on the physiological timescale, using calcium jumps produced by photolysis of caged calcium. The results reveal three activation states of the thin filament in the intact filament lattice of a muscle cell that are analogous to those proposed previously from studies on isolated proteins. We characterize the rates of the transitions between these states in relation to thick filament mechano-sensing and show how thin- and thick-filament-based mechanisms are coupled by two positive feedback loops that switch on both filaments to achieve rapid cooperative activation of skeletal muscle.

## Significance

Contraction of skeletal muscle is triggered by regulatory structural changes in the thin filaments following calcium binding to troponin. Structural changes in the thick filaments control the availability of myosin motors for actin interaction and the strength and speed of contraction. Here, we elucidate the coupling between thin and thick filament regulatory mechanisms in demembranated fibers from mammalian skeletal muscle in near-physiological conditions, both in the steady state and on the millisecond timescale following a calcium jump. We show that physiological activation of skeletal muscle depends on two positive feedback loops, involving mechano-sensing by the thick filament and myosin-sensing by the thin filament. The rapid activation of skeletal muscle following electrical stimulation depends on the coordinated activation of both filaments.

The contraction of skeletal muscle is generated by the relative sliding of actin-containing thin filaments and myosin-containing thick filaments driven by myosin motors transiently interacting with actin and is triggered by calcium-dependent regulatory structural changes in the thin filaments. In resting muscle, the regulatory proteins troponin and tropomyosin hold the thin filament in the blocked state by masking the myosin-binding sites on actin (1–3). Following electrical stimulation, calcium ions are released from intracellular stores and bind to the regulatory head of troponin in the N-lobe of troponin C (TnC), triggering dynamic changes in the structure of the troponin complex which lead to the azimuthal motion of tropomyosin away from its inhibitory position on the thin filament, partially uncovering the myosin-binding sites on actin (2, 4). However, the myosin motors are not directly available for interaction with actin because they are locked in helical tracks on the surface of the thick filament in a folded conformation called the interacting-heads motif (IHM) (5–8), likely associated with the “superrelaxed” state of myosin (9). Recently, it has been proposed that the thick filament acts as a mechano-sensor that modulates the number of active myosin motors according to the filament stress and independently of the regulatory state of the thin filament (7, 10). According to this mechano-sensing hypothesis, derived from X-ray diffraction studies in amphibian (7) and mammalian (11) muscle, the activation of the folded motors on the thick filament is triggered by the force generated by a pool of “constitutively ON” motors that are immediately available for interaction with actin.

The kinetics of the regulatory structural changes in thick (12) and thin (4) filaments during an isometric contraction have been characterized by time-resolved X-ray diffraction in amphibian skeletal muscle at 4 °C. According to those studies, the motion of tropomyosin on the thin filament during contraction is faster than the rise in force (4) and faster than the speed at which the myosin motors are released from the thick filament surface (12), suggesting that the rate of force generation is mainly limited by the activation of the thick filament. A recent X-ray study on intact mouse EDL muscle at 28 °C also suggested that the rate of the regulatory structural changes in the thick filament controls the dynamics of force generation in twitch and tetanic contractions (13).

However, both the kinetics and the calcium dependence of thin filament activation and the interaction between thin and thick filament regulation in mammalian skeletal muscle have not been characterized in near-physiological conditions. Previous kinetic studies of structural changes in tropomyosin (14) and troponin (15) in demembranated fibers from rabbit psoas muscle activated by photolysis of caged calcium were carried out at much lower temperature (range 4 to 11 °C). Nearly all the previous studies of regulation in demembranated fibers using steady-state calcium titrations, including those with probes on troponin (16), were also carried out at low temperature, conditions in which the fibers can be activated repeatedly with little decline in force production.

Unfortunately, the conditions used for those previous studies on demembranated fibers from mammalian muscle inadvertently excluded the thick-filament-based regulatory mechanisms because the OFF state of the thick filament is lost in the conditions that were used. Both the low temperature and the increased myofilament lattice spacing in demembranated fibers disrupt the physiological folded-helical conformation of the myosin motors in the relaxed thick filament (17–19). The native structure of the thick filaments in intact resting mammalian muscle at physiological temperature is preserved only in demembranated fibers at a temperature of 26 °C and above and when the physiological lattice spacing has been restored by osmotic compression (19). The mechanosensing transition in the thick filament is preserved during activation at steady [Ca^2+^] in these conditions (10).

Here, we elucidated the mechanisms of activation of thin and thick filaments and their coupling in skeletal muscle in near-physiological conditions which preserve both thin and thick filament-based regulatory mechanisms. We used time-resolved polarized fluorescence from bifunctional rhodamine probes in the regulatory domains of TnC and in the regulatory light chain (RLC) of myosin, exchanged into demembranated muscle fibers, to measure dynamic changes in the conformation of troponin and of the myosin motors, respectively, during contractions at steady [Ca^2+^] or triggered by [Ca^2+^]-jumps, with high signal-to-noise and temporal resolution. Our results show that, in contrast with some previous models of thin and thick filament regulation, the thin filament is only partially activated by calcium and requires force-generating myosin motors bound to actin to be fully switched on, whereas the thick filament is activated by a mechano-sensing mechanism. We propose a dual-filament model of regulation of muscle contraction in which two positive feedback loops triggered by the calcium transient couple the activation of thin and thick filaments and account for the rapid activation of skeletal muscle following electrical stimulation.

## Results and Discussion

### The Coupling between Regulatory States of Thin and Thick Filaments in Skeletal Muscle at Steady [Ca^2+^].

To investigate the steady-state calcium dependence of the regulatory structural changes in the thin filaments, we introduced bifunctional rhodamine probes on the C-helix (TnC-C probe) or E-helix (TnC-E probe) of TnC in demembranated skeletal muscle fibers. The TnC-C probe is in the N-lobe of TnC, which forms the regulatory head of troponin, and it is mainly sensitive to the opening of the lobe induced by calcium binding to the regulatory sites (15, 16). The TnC-E probe is in the C-lobe of TnC, and it reports changes in the orientation of the IT arm of troponin with respect to the filament axis induced either by calcium or by myosin motors displacing the tropomyosin/troponin complex from its inhibitory position (15, 16). Changes in the conformation of the myosin motors on the thick filament were measured using a bifunctional rhodamine probe on the E-helix in the C-lobe of the RLC of myosin (RLC-E probe), which is sensitive to the transition from the parallel/folded to the perpendicular/active conformation of the myosin motors (10, 17). Probe orientations were measured in situ in terms of the order parameter <*P*_2_>, which would take values 1 and −0.5 for probe orientations parallel and perpendicular to the filament axis, respectively (20).

In relaxed (pCa = 9) demembranated muscle fibers, the folded helical conformation of the myosin motors on the thick filament typical of intact muscle at rest is preserved only at temperatures higher than 26 °C and at physiological filament lattice spacing in the presence of the osmotic agent Dextran T-500 (5% w/v) (17, 19), whereas the conformation of troponin on the thin filament is only slightly sensitive to temperature and osmotic compression (*SI Appendix*, Fig. S1). Therefore, we activated the muscle fibers using steady [Ca^2+^] at 26 °C in the presence of Dextran, in order to preserve the physiological regulatory mechanisms in both thin and thick filaments.

**Fig. 1.**
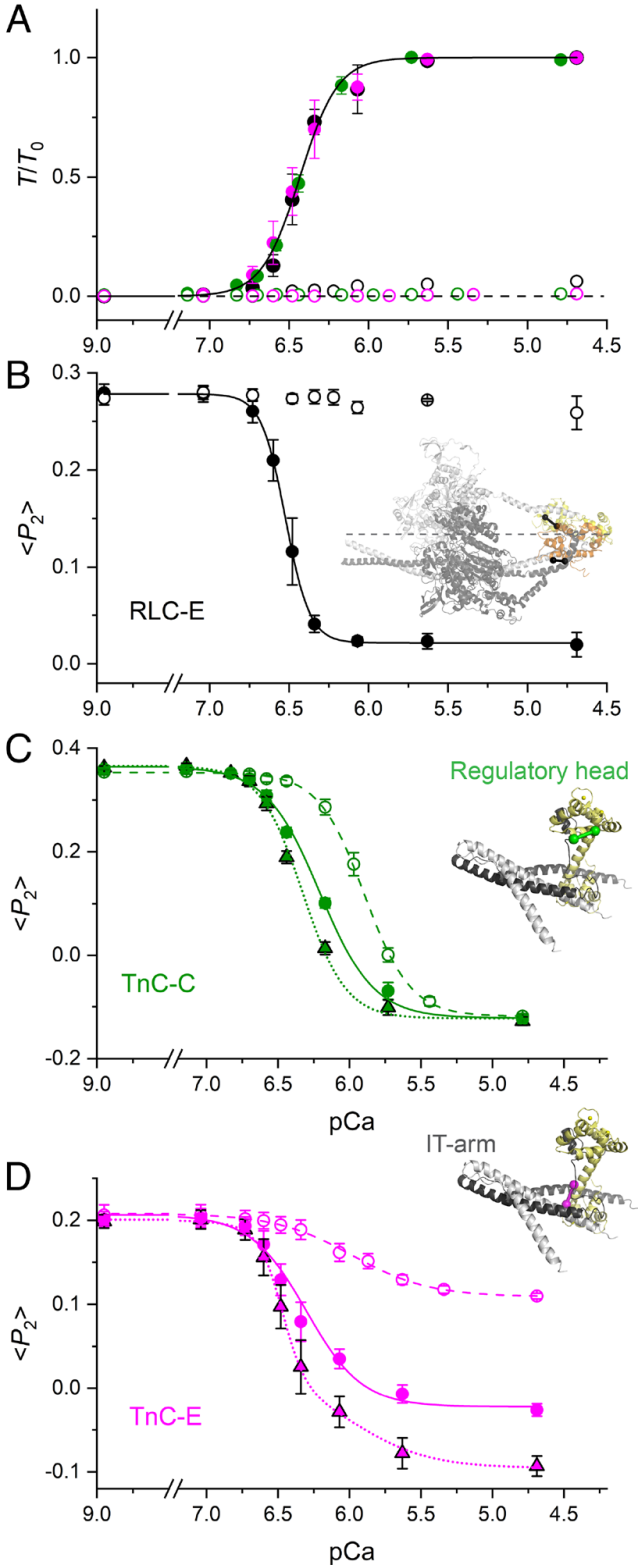
Steady-state calcium dependence of force and of the orientation of TnC and RLC probes in skeletal muscle fibers at near-physiological temperature and lattice spacing. (*A*) Dependence of steady-state force (mean ± SE) normalized to the force at maximal [Ca^2+^] (*T*_0_=248 ± 10 kPa, mean ± SE, n = 15 fibers) on pCa (=−log_10_[Ca^2+^]), in single muscle fibers exchanged with the RLC-E probe (black; n = 5 fibers), TnC-C probe (green; n = 5 fibers), and TnC-E probe (magenta; n = 5 fibers) at 26 °C with 5% Dextran T-500 (w/v) and sarcomere length 2.45 µm, in the absence (filled symbols) and in the presence (empty symbols) of 25 µM blebbistatin. Calcium dependence of <*P*_2_> (mean ± SE) for the RLC-E probe (*B*, data from ref. 10), TnC-C probe (*C*; n = 5 fibers), and TnC-E probe (*D*; n = 5 fibers) in the absence (filled circles) and in the presence (empty circles) of 25 µM blebbistatin. Triangles in *C* and *D* represent <*P*_2_> calculated for the TnC probes in the A-band of the sarcomere in the absence of blebbistatin. Solid and dashed lines in *C* and *D* are single-Hill curves fitted to the data in the absence or in the presence of 25 µM blebbistatin, respectively. Dotted lines in *C* and *D* are single- and double-Hill curves, respectively, fitted to <*P*_2_> in the A-band in the absence of blebbistatin. The orientation of the RLC-E probe (black) in the RLC in the blocked (orange) and free (yellow) head in the human β-cardiac myosin IHM [PDB 5TBY (21), essential light chains not shown for clarity] with respect to the filament axis (dashed line) is shown in *B*. The positions of the TnC-C probe in the regulatory head and of the TnC-E probe in the IT arm in the Ca^2+^-saturated troponin core structure [PDB 1J1D (22)] are shown in *C* and *D*, respectively. Graphical representations of RLC and TnC probe orientations were generated using the PyMOL Molecular Graphic System (DeLano Scientific, LLC). pCa values for fibers exchanged with labeled TnC-C were corrected for the shifts due to probe conjugation as described in *Materials and Methods*.

The calcium dependence of force in these conditions was described by a Hill curve with calcium sensitivity (pCa_50_) 6.43 ± 0.01 and cooperativity (*n*_H_) 3.64 ± 0.20 (mean ± SEM, n = 15; Fig. 1*A* and Table 1). <*P*_2_> for the RLC probe decreased more steeply with increasing calcium concentration, signaling the release of myosin motors from the folded conformation with a calcium sensitivity and a cooperativity higher than those of force (Fig. 1*B* and Table 1) (10). <*P*_2_> for both TnC probes also decreased with increasing calcium concentration (Fig. 1 *C* and *D*), indicating a more perpendicular orientation of the probe with respect to the filament axis but with pCa_50_ and *n*_H_ *lower* than those of force (Table 1).

**Table 1.** Calcium dependence of force and <*P*_2_> for TnC and RLC probes

		A	B_pCa9_	*pCa*_50_	*n_H_*
Force		1 (248 ±10 kPa)	0	6.43 ± 0.01	3.64 ± 0.20
<*P*_2_> RLC-E		−0.257 ± 0.002	0.278 ± 0.001	6.523 ± 0.002^^*^^	5.67 ± 0.15^^*^^
<*P*_2_> TnC-C		−0.486 ± 0.009	0.364 ± 0.004	6.21 ± 0.02^^*^^	2.31 ± 0.19^^*^^
<*P*_2_> TnC-C_Bleb_		−0.471 ± 0.002	0.353 ± 0.001	5.90 ± 0.01^^†^^	2.69 ± 0.09^^†^^
<*P*_2_> TnC-C_A-band_		0.488 ± 0.009	0.366 ± 0.003	6.33 ± 0.02	2.89 ± 0.22
<*P*_2_> TnC-E		−0.229 ± 0.009	0.206 ± 0.007	6.30 ± 0.04^^*^^	2.22 ± 0.31^^*^^
<*P*_2_> TnC-E_Bleb_		−0.099 ± 0.002	0.208 ± 0.002	5.98 ± 0.02^^†^^	1.61 ± 0.09^^†^^
<*P*_2_> TnC-E_A-band_	M	−0.187 ± 0.010	0.201 ± 0.002	6.47 ± 0.01	5.26 ± 0.68
	Ca	−0.109 ± 0.011	–	*5.98*	*1.61*

Parameters of Hill curves fitted to force and <*P*_2_> data in Fig. 1 in the presence or absence of blebbistatin. <*P*_2_> TnC-E_A-band_ was better fitted with a double-Hill curve, according to the Bayesian information criterion test (*Materials and Methods* and *SI Appendix*, Fig. S2), which includes myosin (M) and calcium (Ca) dependent components. The pCa_50_ and n_H_ for the calcium-dependent component (Ca) were constrained to the values (in italic) determined in the presence of blebbistatin (<*P*_2_> TnC-E_Bleb_). Errors are SE estimated by the fit.

^*^P < 0.05 when comparing pCa50 and nH of force and <*P*_2_> using a paired *t*-test.

^†^*P* < 0.05 when comparing pCa50 and nH of <*P*_2_> in the absence or presence of blebbistatin using a paired *t*-test.

To separate the effects of force and Ca^2+^ on the structure of thin and thick filaments, we determined the calcium dependence of <*P*_2_> for TnC and RLC probes in the presence of 25 µM blebbistatin. Blebbistatin abolished both the active force (Fig. 1*A*; open symbols and dashed line) and the changes in RLC orientation (Fig. 1*B*), stabilizing the folded conformation of myosin on the thick filament (17), and decreased the calcium sensitivity of <*P*_2_> for both TnC probes by 0.3 pCa units (Fig. 1 *C* and *D* and Table 1). This result indicates that the attachment of force-generating myosin motors to actin sensitizes both the regulatory head and the IT arm of troponin to calcium. However, blebbistatin did not affect the amplitude of the calcium-dependent change in <*P*_2_> for the TnC-C probe (Fig. 1*C*), but it greatly decreased that for the TnC-E probe (Fig. 1*D*). At saturating calcium concentration (pCa 4.7), the conformation of the regulatory head of troponin is insensitive to myosin attachment, but the orientation of the IT arm depends on both myosin attachment and calcium binding, with myosin making a much larger contribution.

These myosin-dependent changes in troponin orientation occur predominantly in the A-band region of the sarcomere, where the thin filaments overlap with thick filaments. At the sarcomere length used here (2.45 µm), about 2/3 of the troponin/tropomyosin complexes are in the A-band, and the remaining 1/3 are in the I-band, where they cannot interact with myosin. We therefore estimated <*P*_2_> for the TnC probes in the A-band from the average probe orientation at each [Ca^2+^] under the assumption that the calcium sensitivity and cooperativity of the TnC probes in the I-band are the same as that in the presence of blebbistatin (*Materials and Methods*) and are not influenced by the structural changes in the thin filament induced by myosin in the A-band.

The calcium dependence of <*P*_2_> for TnC-C in the A-band (Fig. 1*C*, triangles; Table 1) was characterized by a Hill curve with pCa_50_ 0.4 pCa units higher than that in the I-band, whereas *n*_H_ was not significantly different (Table 1, paired *t* test *P = *0.32), indicating that force-generating myosin motors sensitize the N-lobe of TnC to calcium, without affecting the cooperativity of its structural change or the conformation at saturating [Ca^2+^].

The calcium dependence of <*P*_2_> for the TnC-E probe in the A-band (Fig. 1*D*, triangles) was not well fit by the Hill equation (*SI Appendix*, Fig. S2). We therefore tested the hypothesis that its orientation change is the sum of a purely calcium-dependent component (TnC-E_Ca), like that observed in the presence of blebbistatin, and a myosin-dependent component (TnC-E_M). The resulting double Hill equation (Fig. 1*D*, dotted line) gave a much better fit to the <*P*_2_> data, as confirmed by the Bayesian information criterion (BIC) test (Table 1, *SI Appendix*, Fig. S2, and *Materials and Methods*), and indicated that the TnC-E_M component accounts for ∼75% of the total calcium-dependent orientation change. Moreover, the M component has a calcium sensitivity (pCa_50 _= 6.47) not significantly different from that of force (Table 1, paired *t* test *P = *0.89) and a high cooperativity (*n*_H_ = 5.02; Table 1).

The calcium dependence of the orientation change of the TnC probes described above is different in several respects from that measured previously at 11 °C in the absence of Dextran (16). Under those conditions, the parallel-to-perpendicular transition of the myosin motors in the thick filament seen during activation at higher temperature is abolished (*SI Appendix*, Fig. S3). Moreover, at low temperature, the TnC-C probe is insensitive to the attachment of myosin motors to actin, and the cooperativity of orientation changes in the TnC-E probe is much lower (*n*_H_ ∼2) and independent of myosin-attachment to actin (16). These results show that both the regulatory structural changes in myosin and the contribution of myosin to the activation of the thin filament are severely attenuated in demembranated muscle fibers at low temperature with an expanded lattice, the conditions used in nearly all previous studies of regulation in mammalian muscle.

These results highlight the distinct regulatory roles of the N-lobe of TnC and the IT arm of troponin in skeletal muscle in near-physiological conditions. The TnC N-lobe acts as the calcium sensor of the thin filament, and its extreme OFF and ON conformations are independent of the binding of myosin motors to the thin filament. However, the sensitivity of the TnC N-lobe to calcium is increased by attachment of myosin motors to actin, an effect that is absent at low temperature (16) and might be mediated by the displacement of TnI from actin induced by myosin, which in turn increases the calcium sensitivity of TnC (23). In contrast, the IT arm of troponin integrates the calcium signal from the N-lobe of TnC with the myosin signal from the thick filament, coupling the regulatory pathways of the thin and thick filaments. In particular, the myosin-dependent component of the orientation change of the IT arm (TnC-E_M) tracks the binding of force-generating myosin motors to actin, probably by coupling the azimuthal position of tropomyosin to rotation of the IT arm. We conclude that the thin filaments in skeletal muscle are only partially activated by calcium and that full activation and cooperativity require binding of force-generating myosin motors, consistent with recent mechanical evidence that the cooperativity of thin filament activation depends on the force per myosin motor (24). This conclusion extends the previous concept of three regulatory states of the thin filament corresponding to three azimuthal positions of tropomyosin, the blocked, closed, and open states, inferred from studies on the isolated proteins (25) to the intact filament lattice in near-physiological conditions.

**Fig. 2.**
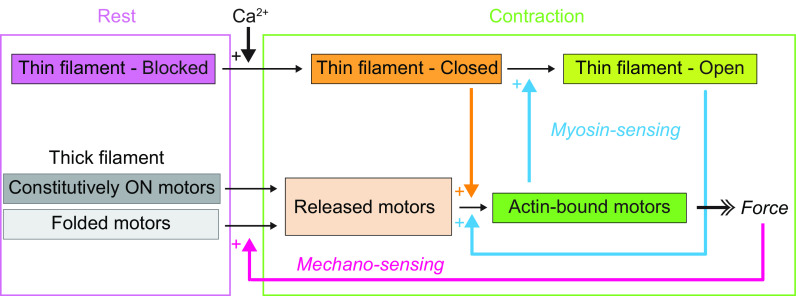
Dual-filament mechanism of activation in skeletal muscle. Schematic diagram of the two positive feedback loops controlling the activation of thin (myosin-sensing) and thick (mechano-sensing) filaments triggered by calcium. See main text for details.

Our results lead to a dual-filament model of the activation of contraction in skeletal muscle, in which the steady force is controlled by two positive feedback loops in thin and thick filaments triggered by calcium (Fig. 2). In resting muscle (Fig. 2, purple rectangle), the thin filament is blocked by tropomyosin masking the myosin-binding sites on actin, while in the thick filament, most of the myosin motors are folded with few constitutively ON motors released from the filament surface. During contraction (Fig. 2, green rectangle), Ca^2+^ ions partially activate the thin filament (thin filament-closed state) to which the constitutively ON myosin motors can attach (actin-bound motors). The force generated by the actin-bound motors triggers the release of the folded myosin motors in the thick filament, increasing the fraction of actin-bound motors and initiating a positive mechano-sensing feedback loop (Fig. 2, magenta) which accounts for the highly cooperative activation of the thick filament. The increase in the number of actin-bound motors further activates the thin filament (thin filament-open state, Fig. 2) allowing more myosin motors to bind to actin and triggering a positive myosin-sensing feedback loop in the thin filament (Fig. 2, blue), which couples the regulatory state of the thin filament to that of the thick filament at the steady state.

### The Dynamics of Thin and Thick Filament Activation during Isometric Contraction of Skeletal Muscle.

Under physiological conditions, the contraction of fast-twitch skeletal muscle is triggered by a large and rapid increase in [Ca^2+^] to saturating levels, but the dynamics of force generation is much slower than the changes in [Ca^2+^] (26). How do the two positive feedback loops in the activation of thin and thick filaments control the force output of muscle during a transient physiological activation? To answer this question, we measured the time course of the orientation changes of the TnC and RLC probes with 0.12-ms time resolution in single demembranated muscle fibers activated by photolysis of caged calcium (NP-EGTA) in conditions of temperature and lattice spacing (T = 26 °C, 5% w/v Dextran T-500) in which the two positive feedback loops described above are preserved. The photolysis of caged calcium triggers the release of calcium ions with a rate constant of ∼70,000 s^−1^ (27), rapidly raising [Ca^2+^] to saturating levels throughout the volume of the muscle fiber. This method of activation of the muscle fiber avoids the temporal delays and the spatial gradients of calcium concentration associated with physiological calcium release in intact muscle cells (28).

**Fig. 3.**
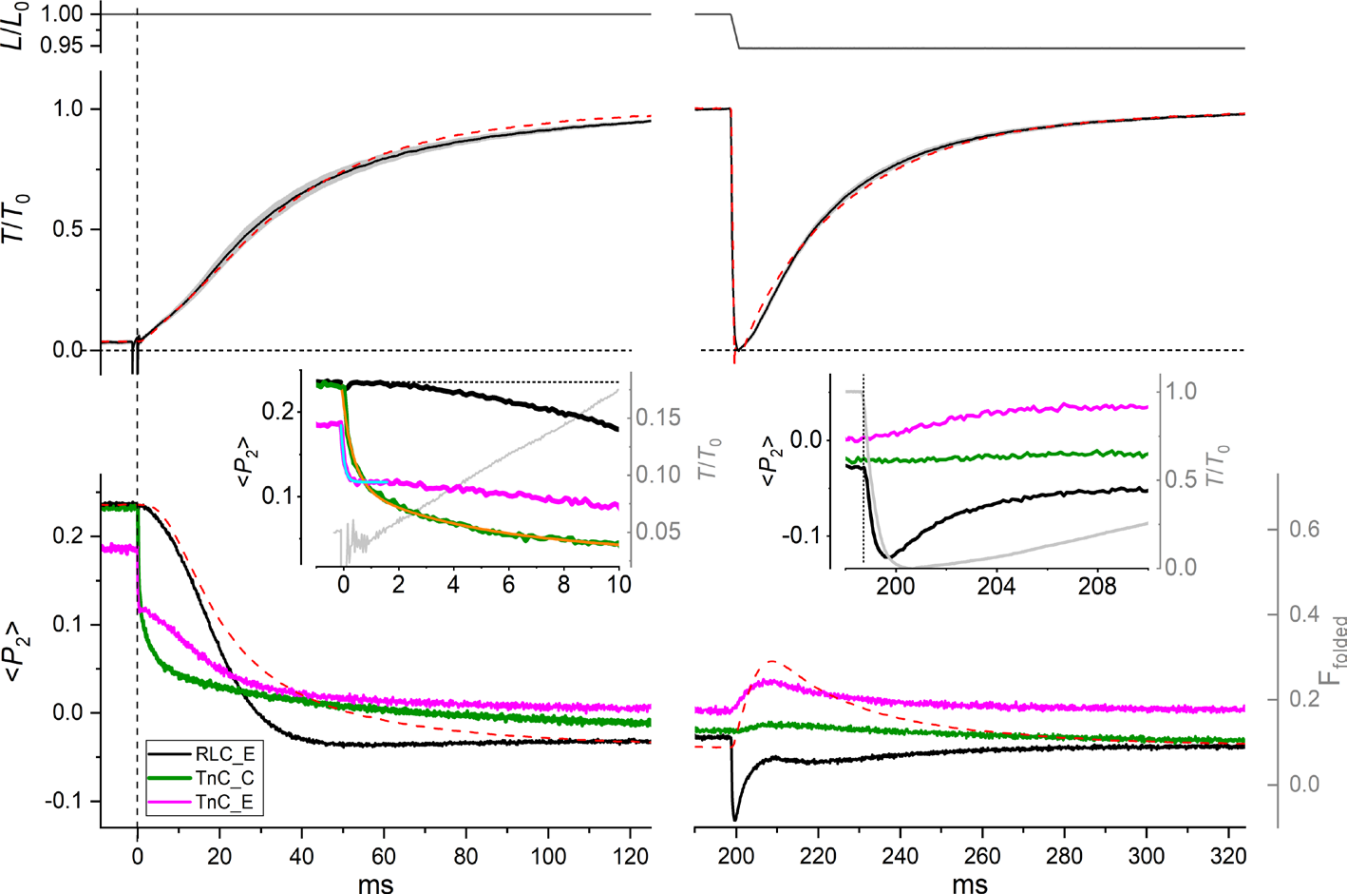
Structural dynamics of TnC and RLC in muscle fibers activated by photolysis of caged calcium. *Upper* panel, fiber length *L* relative to the initial length *L*_0_. *Middle* panel, time course of force (average, black; SE, gray, n = 15 fibers) relative to the value at the plateau of contraction *T*_0_ (=262 ± 10 kPa, mean ± SE, n = 15 fibers) after the UV photolysis of caged calcium at time zero (vertical dashed line). The horizontal dashed line marks the zero force. Red dashed line, model simulation of the time course of force (see main text). *Lower* panel, average time course of the order parameter <*P*_2_> for the RLC-E probe (black, n = 7 fibers), TnC-C probe (green, n = 4 fibers), and TnC-E probe (magenta, n = 4 fibers). Red dashed line, model simulation of the time course of the fraction of folded myosin motors (F_folded_) on the thick filament. *Left* *Inset*, force (gray) and <*P*_2_> for RLC and TnC probes in the first 10 ms after photolysis; cyan line, single-exponential fit to <*P*_2_> for TnC-E probe; orange line, three-exponential fit to <*P*_2_> for TnC-C probe (*SI Appendix*, Table S1); dotted line, relaxed value of <*P*_2_> for the RLC-E probe. *Right* *Inset*, responses of force (gray) and <*P*_2_> for RLC and TnC probes to ramp shortening. T = 26 °C, 5% (w/v) Dextran T-500, initial sarcomere length 2.4 µm.

After photolysis, force increased with a delay of 1 ms (Fig. 3, *Inset*) and with an overall half-time of 29 ± 2 ms (mean ± SE, n = 15; *SI Appendix*, Table S1), accompanied by sarcomere shortening of ∼5% (*SI Appendix*, Fig. S4). <*P*_2_> for both the TnC-C and TnC-E probes showed large decreases within 5 ms of photolysis (Fig. 3, green and magenta traces, respectively). Multiexponential fitting of <*P*_2_> for the TnC-C probe detected two components, together accounting for ∼72% of the total <*P*_2_> change (Fig. 3, *Inset*, orange trace), with rate constants of ∼3,800 s^−1^ and ∼370 s^−1^, two and one orders of magnitude faster than force generation, respectively, and a third component with a rate constant of ∼20 s^−1^ (*SI Appendix*, Table S1). The fastest phase provides a direct measure of the rate constant for binding of calcium ions to the regulatory Ca^2+^ sites in the N-lobe of TnC in situ and is consistent with the diffusion-limited rate of Ca^2+^ association to TnC measured in solution (29). The second phase has a rate similar to that of the azimuthal motion of tropomyosin around the actin filament, as determined from the time course of the intensity of the second actin layer line in X-ray diffraction studies in amphibian muscle (t_50 _= ∼5 ms at 22 °C) (4) and in demembranated fibers from rabbit psoas muscle activated by caged-calcium photolysis (r = 113 s^−1^ at 4 °C) (14), taking into account the lower temperatures used in those studies. The third phase is likely to be associated with the binding of Ca^2+^ to the Ca^2+^-Mg^2+^ sites in the C-lobe of TnC, and its rate constant is about 100 times slower than that for the binding of calcium ions to the regulatory sites in the N-lobe, in agreement with measurements on isolated TnC (29).

The caged-calcium approach allowed us to kinetically separate the calcium- and myosin-dependent structural changes in the IT arm of troponin and compare them with those estimated from the steady-state calcium dependence. Calcium binding to TnC induced a fast change in <*P*_2_> for the TnC-E probe with a rate constant of ∼7000 s^−1^ (Fig. 3, *Inset*, cyan trace; *SI Appendix*, Table S1), not significantly different from that of the fast phase of the TnC-C probe (*t* test *P* = 0.13), which accounted for 36% of the total <*P*_2_> change, similar to the fractional amplitude of the calcium-dependent component estimated from the steady-state calcium dependence (Fig. 1*D* and Table 1). These results therefore show that the calcium-binding signal from the N-lobe of TnC is transmitted to the IT arm with no measurable delay on the submillisecond time scale. A slower phase in the orientation change of the TnC-E probe had a time course slightly faster than that of force generation (Fig. 3, magenta), although its half-time, 17 ± 2 ms (mean ± SE, n = 4; *SI Appendix*, Table S1), was not significantly different from that of force (paired *t* test *P* = 0.12). The fractional amplitude of this phase calculated for the probes in the A-band is ∼70%, similar to the amplitude of the TnC-E_M component measured at the steady state (Fig. 1*D* and Table 1). The kinetics of TnC-E_M tracks myosin binding to actin and is consistent with that of the force-dependent component of the azimuthal motion of tropomyosin observed by X-ray diffraction in muscle fibers activated by caged calcium (14). Therefore, we conclude that TnC-E_M is associated with the transition of the thin filament to the open state in the myosin-sensing feedback loop of thin filament activation (Fig. 2).

The structural dynamics of troponin described above are different in several respects from those seen in a previous study at 11 °C in the absence of Dextran (15). Each component of the orientation change of the TnC-C probe at 26 °C is about three times faster than that at 11 °C. In contrast, the overall time course of the orientation change of the TnC-E probe was *slower* at 26 °C than that at 11 °C, being 90% complete at 25 ms after photolysis, ∼15 ms slower than at 11 °C (*SI Appendix*, Fig. S5). This unexpected result may be partially explained by the fact that the amplitude of the myosin-dependent change in orientation of the TnC-E probe is reduced to ∼20% at low temperature (15), consistent with the smaller contribution of myosin to the activation of the thin filament at low temperature deduced from experiments at steady [Ca^2+^] (16), as discussed above. Moreover, the myosin motors are already perpendicular to the filament axis at low calcium at 11 °C in the absence of Dextran (*SI Appendix*, Fig. S3) and do not undergo large conformational changes during activation by photolysis of caged calcium (*SI Appendix*, Fig. S5), consistent with the hypothesis that the thick filament is already partially switched on in relaxed muscle at low temperature (19). In contrast, at 26 °C, the thin filament requires the attachment of myosin motors to actin for full activation, but the motors are not immediately available because they first have to be released from their folded conformation on the surface of the thick filament. The kinetics of activation of the myosin motors, estimated from the time course of <*P*_2_> for the RLC-E probe, was much slower than that of the TnC probes, and it was sigmoidal in shape, with an initial lag of ∼3 ms (Fig. 3, black). This time course is consistent with the idea that the initial small and slow rise of force is generated by a population of constitutively ON motors that can immediately interact with an activated thin filament, whereas the later and larger component requires the release of a larger pool of myosin motors from the folded OFF state. The overall half-time of the transition was 18 ± 1 ms (mean ± SE, n = 7; *SI Appendix*, Table S1), so release of myosin motors from the folded state was almost synchronous with the myosin-related phase of the TnC-E signal (Fig. 3, magenta; *SI Appendix*, Table S1, *t* test *P* = 0.78). Both these signals have a half-time that is about 10 ms faster than force generation itself. The correspondence between the time courses of RLC-E and myosin–related TnC-E signals shows that the associated structural changes are coupled both in their steady-state calcium dependence (Table 1) and in their dynamics on the timescale of muscle activation.

The <*P*_2_> change for the RLC probe was complete at ~40 ms after photolysis, indicating that the myosin motors on the thick filament were fully activated when the force was only ∼65% of the maximal isometric force. The time course of activation of the myosin motors measured here by fluorescence polarization is consistent with that measured by X-ray diffraction in intact EDL muscles of the mouse during tetanic stimulation at 28 °C (13). In that study, the release of myosin motors from the folded state, signaled by the increase in the X-ray interference parameter *L*_M3_, had a half-time of ∼11 ms, leading force generation by ∼6ms (half-time ∼17 ms), and it was complete when force was 60% of the maximum value (13). The kinetics of force generation and of activation of the myosin motors in intact mouse EDL muscle are similar to those measured here in demembranated fibers from rabbit psoas muscle. We conclude that the physiological mechanism of thick filament activation and its dynamic relation with force generation are preserved in isolated demembranated fibers activated by photolysis of caged calcium in the conditions of the present experiments.

**Fig. 4.**
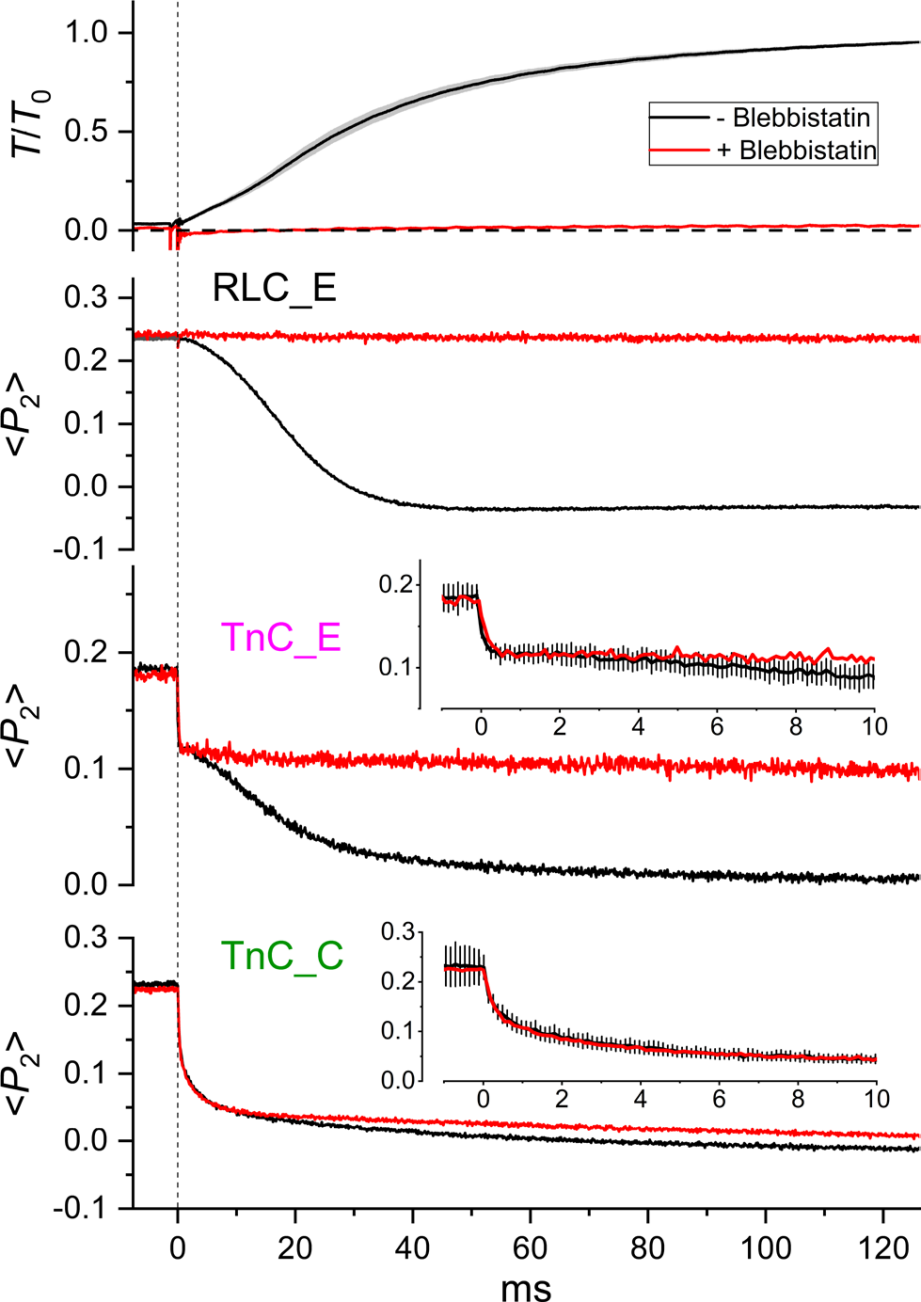
Structural dynamics of TnC and RLC during activation in the presence of blebbistatin. Time courses of force, relative to the value at the plateau of contraction *T*_0_ (=262 ± 10 kPa, mean ± SE, n = 15 fibers), and of <*P*_2_> for RLC and TnC probes in the absence (black; as in Fig. 2) and in the presence of 25 µM blebbistatin (red; n = 1 fiber for each probe). The horizontal dashed line marks the zero force; the vertical dashed line marks the UV photolysis of caged calcium at time zero. *Insets*, time course of <*P*_2_> for TnC probes on an expanded time scale in the absence (black, mean ± SE, n = 4 for TnC-C probe and n = 4 for TnC-E probe) and in the presence (red, n = 1) of blebbistatin; T = 26 °C, 5% (w/v) Dextran T-500, initial sarcomere length 2.4 µm.

To further characterize the effect of myosin attachment to actin on the dynamics of these structural changes, we activated a set of muscle fibers by photolysis of caged calcium in the presence of 25 µM blebbistatin (Fig. 4). The amplitude and kinetics of the orientation change of the TnC-C probe and the fast phase of the TnC-E probe were not affected by blebbistatin, whereas the slower phase that we had identified with myosin binding in the absence of blebbistatin on the basis of its kinetics was abolished. We conclude that the dynamics of the structural change in the regulatory head of troponin and those of the fast component of the structural change in the IT arm are insensitive to myosin during activation at maximal [Ca^2+^].

**Fig. 5.**
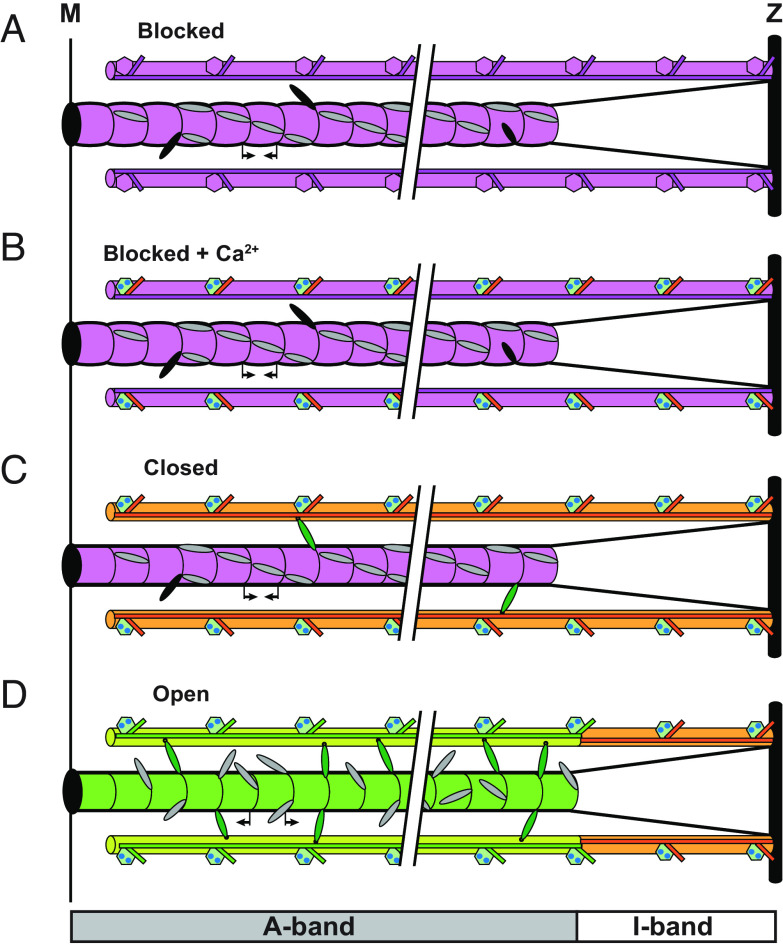
Dynamic dual-filament activation of contractility in skeletal muscle. Each half thick filament extends from the M band and is connected to the Z band (vertical black rectangles) by titin links (black lines). Thin filaments are anchored at the Z band. (*A*) In the absence of calcium, the thin filament is in the inhibited or “blocked” state (purple) with tropomyosin (horizontal purple line) masking the myosin-binding sites on actin. The thick filament is in the “locked” state (purple); most of the myosin motors are folded in helical tracks on the filament surface (gray ellipses), but there are a few constitutively ON motors (black ellipses). The thick filament periodicity is short in the absence of filament stress (inward pointing arrows). (*B*) During activation, calcium ions bind to the regulatory head of troponin (green hexagons) triggering a fast rotation of the IT arm of troponin (orange rectangles). The tropomyosin has not moved yet, and the thin filament is in the “blocked+Ca^2+^” state. (*C*) The motion of tropomyosin partially unmasks the myosin-binding sites on actin, and the thin filament is in a partially activated closed state (orange). The constitutively ON motors can attach to actin and generate force (green ellipses), triggering the mechano-sensing activation of the thick filament. (*D*) At the plateau of contraction, the folded myosin motors are released from the filament surface, and the thick filament is fully activated (green). The backbone periodicity of the thick filament has increased (outward-pointing arrows). Myosin motors attached to actin in the filament overlap region of the half-sarcomere (A-band) further displace tropomyosin and induce the fully activated open state of the thin filament (light green), whereas the thin filament at no-overlap in the I-band remains in the closed state (orange).

In summary, the present results elucidate the dynamic coupling between regulatory structural changes in thin and thick filaments in the dual-filament mechanism of force generation in skeletal muscle. During physiological activation, calcium ions saturate the binding sites in the N-lobe of TnC with a rate constant of ∼4,000 to 7,000 s^−1^ triggering a fast reorientation of the IT arm of troponin on the thin filament. In this transient state, the tropomyosin strand remains in the blocking position and therefore the thin filament is in a “blocked+Ca^2+^” state (Fig. 5*B*), which was not resolved during steady-state activation (Fig. 2). Then, tropomyosin moves on the thin filament partially uncovering the actin-binding sites for myosin, and the thin filament is in the closed state (Fig. 5*C*, orange). Constitutively ON motors can then attach to actin and generate force triggering the release of the folded motors from the surface of the thick filament (Fig. 5*C*). The increase in the fraction of motors attached to actin and in force triggers the myosin-sensing and mechano-sensing feedback loops, in thin and thick filaments respectively, and both the thick and thin filaments in the A-band of the sarcomere become fully active (open state; Fig. 5*D*, green).

### Stress-Dependent Activation of the Thick Filament Coupled to Myosin-Dependent Activation of the Thin Filament Controls the Kinetics of Force Generation in Skeletal Muscle.

The results presented above suggest that the rate of force generation following physiological activation might be limited by that of the stress-dependent activation of the myosin motors on the thick filament and by the consequent myosin-dependent activation of the thin filament. To test that hypothesis, we determined the kinetics of force generation after a brief period (∼3 ms) of unloaded shortening applied at the plateau of contraction, which induces detachment of myosin motors from actin. The orientation of the TnC-C probe was almost unaffected by the shortening (Fig. 3, green), consistent with the conclusion from the steady-state measurements that the regulatory head of troponin is insensitive to myosin binding at high calcium concentrations (Fig. 1). <*P*_2_> for the TnC-E probe recovered partially and transiently toward its relaxed value (Fig. 3, magenta), consistent with the expected transient detachment of myosin from actin (15). The shortening induced a fast decrease in <*P*_2_> for the RLC-E probe complete in 1 ms (Fig. 3, black), which reports the synchronized tilting of the light chain domains of actin-attached myosin motors toward more perpendicular orientations with respect to the filament axis (30, 31), followed by the recovery of <*P*_2_> toward its active value. These results indicate that the switching off of thick filaments due to low force (7) and of thin filaments due to myosin detachment is slow on the millisecond timescale. At the end of the 3-ms ramp, when the levels of thin and thick filament activation are still much higher than in the first few milliseconds after photolysis, force redeveloped with an exponential time course with a half-time of 18 ms (Fig. 3 and *SI Appendix*, Table S1), significantly smaller than that of the initial force rise (29 ms, paired *t* test *P* = 5·10^−5^). The difference suggests that the rate of the initial force rise is limited by the intrinsic dynamics of the regulatory structural changes in the thin and thick filaments. The rate of force generation after photolysis at low temperature and in the absence of Dextran (*SI Appendix*, Fig. S5), conditions that largely abolish the mechanosensing transition in the thick filament and the myosin-dependent activation of the thin filament, is similar to that during force redevelopment after shortening in those conditions, consistent with the above conclusion.

The kinetics of force generation after photolysis and after ramp shortening were simulated with a mathematical model in which the regulatory transition of myosin from the folded (OFF) to the active (ON) conformation is linearly dependent on filament stress (32), similar to the load dependence of myosin activation determined with probes on the RLC in muscle fibers (10) (Fig. 3, and *SI Appendix*, *Supplementary Text*). The slower time course of force development after photolysis with respect to that of force redevelopment after shortening could be reproduced in the model with a fraction of 35% of constitutively ON motors on the thick filament (Fig.3, red dashed line; *SI Appendix*, Fig. S6, green trace), consistent with that estimated from the distribution of RLC orientations in the relaxed muscle fiber (17). The simulated time course of the OFF-to-ON transition of the myosin motors reproduced the lag and the dynamics of the conformational change in the RLC during early activation after photolysis, but at higher filament stress the calculated decrease in the fraction of folded motors was slower than the RLC orientation change (Fig. 3). These results suggest that the kinetics of activation of the myosin motors during the early phase of contraction is consistent with a mechano-sensing positive feedback loop triggered by the filament stress, which limits the kinetics of force generation after photolysis. However, the hypothesis of a linear dependence of force on the activation of the folded myosin motors does not explain the rapid activation of the motors, which is complete at ∼60% of the maximal force, suggesting that the activation of the thick filament is highly cooperative, as observed in the calcium dependence at the steady state (Fig. 1*B*), and therefore nonlinearly dependent on filament stress.

**Fig. 6.**
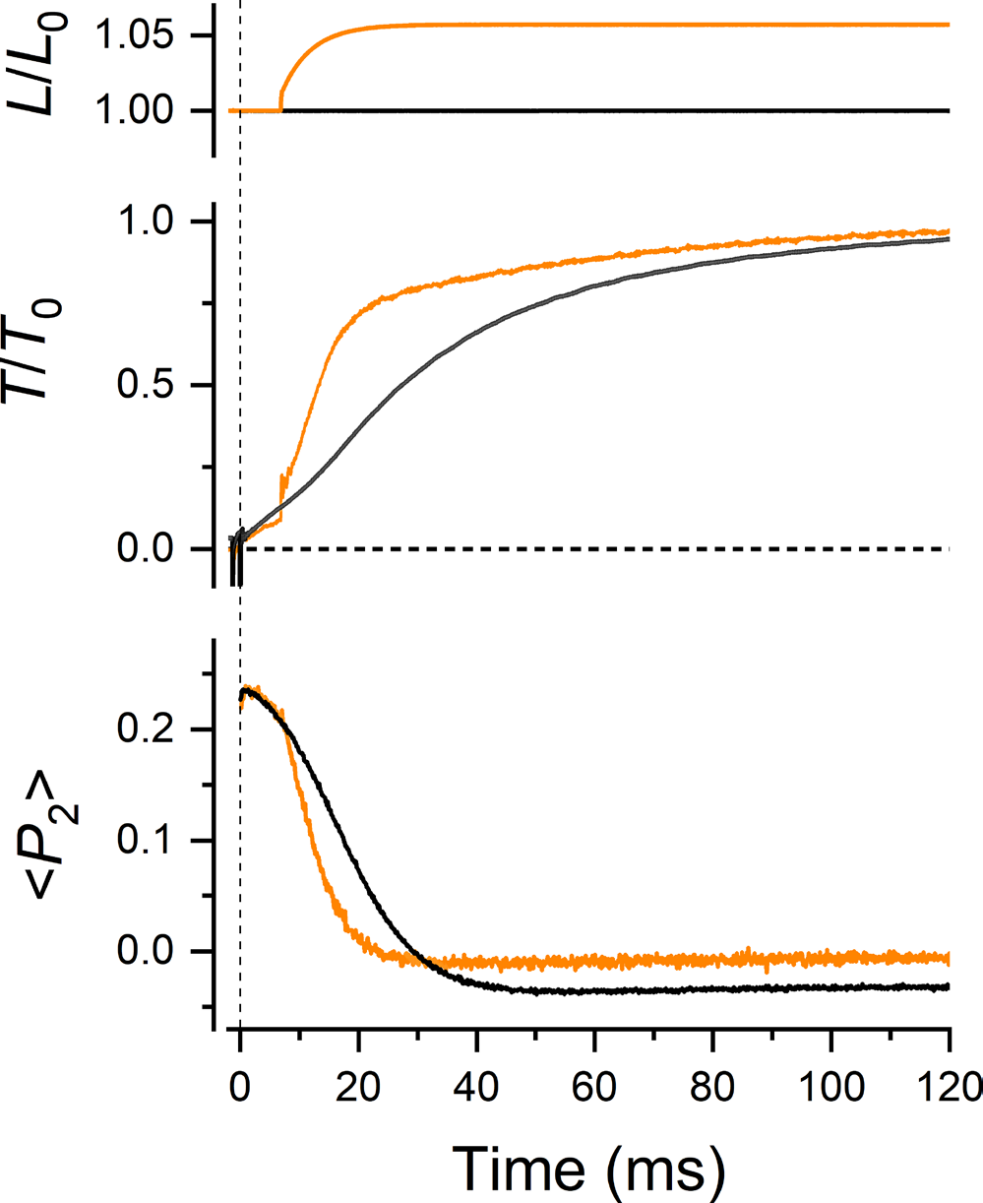
Stretch-induced changes in the dynamics of activation of the myosin motors on the thick filament. Time courses of fiber length (relative to the initial length *L*_0_), force (relative to the value at the plateau of contraction *T*_0_ = 262 ± 10 kPa, mean ± SE, n = 15 fibers) and <*P*_2_> for the RLC-E probe after caged-calcium photolysis at time zero (vertical dashed line) in isometric conditions (black, as in Fig. 2) and with stretch applied to the muscle fiber at 7 ms after the photolysis (orange). The horizontal dashed line marks the zero force. T = 26 °C, 5% (w/v) Dextran T-500, initial sarcomere length 2.4 µm.

To further test the hypothesis that the stress-dependent activation of the thick filament is the rate-limiting step in the kinetics of force generation, we studied the effect of increasing the external load on the thick filament during early activation on the dynamics of thick filament activation and force generation. A rapid stretch of 1% *L*_0_ in 0.3 ms followed by a 5% *L*_0_ exponential lengthening with a time constant of ∼6 ms (Fig. 6, orange trace) was applied 7 ms after photolysis, when force is only 5% of the maximum and most of the myosin motors are still inhibited in the folded conformation on the thick filament. Such a mechanical protocol induces the elongation of the titin spring connecting the thick filament tips to the sarcomere ends (Fig. 5, black lines), increasing the stress on the thick filament during early activation. <*P*_2_> for the RLC-E probe, signaling the release of folded myosin motors on the thick filament, decreased faster than that in isometric condition, and it was accompanied by a much faster increase in force (Fig. 6). This result shows that the kinetics of activation of the myosin motors on the thick filament and the kinetics of force generation in skeletal muscle can be accelerated by increasing the external load applied to the muscle during contraction, as expected from the mechanosensing hypothesis.

### Implications of the Dual-Filament Model for the Regulation of Muscle Contraction.

Our results show that force in skeletal muscle is controlled by a dual-filament mechanism which dynamically couples the regulatory mechanisms in the thin and thick filaments. In demembranated mammalian muscle, this mechanism is preserved only at a temperature of 26 °C or above when the filament lattice is osmotically compressed to match the value in intact resting muscle. At lower temperature with an expanded filament lattice, the mechanosensing transition in the thick filament and its coupling to the myosin-dependent activation of the thin filament are largely abolished, explaining the fact that the dual filament mechanism was not detected in previous studies of thin filament activation (14–16). Moreover, the mechanosensing model of thick filament activation derived from X-ray diffraction studies of intact amphibian (7) and mammalian muscle (11) made the simplifying assumption, in the absence of parallel measurements of thin filament activation in the same protocols, that the thin filament is fully switched on by calcium alone. In contrast with those previous models, the present results show that calcium binding to troponin only partially activates the thin filament, at a speed 100 times faster than the kinetics of force generation. The partial activation of the thin filament triggered by calcium enables the mechano-sensing transition in the thick filament controlling the stress-dependent release of myosin motors from the folded state, which are then available to bind to actin and further activate the thin filament. Mechano-sensing in the thick filament and myosin-sensing in the thin filament constitute two positive feedback loops in the activation of thick and thin filaments (Fig. 2), respectively, that allow skeletal muscle to achieve rapid and cooperative activation following electrical stimulation. In conclusion, our results show that both the steady-state and dynamic mechanisms of force generation in skeletal muscle depend on the coordinated activation of thin and thick filaments, in a dual-filament paradigm of muscle regulation.

## Materials and Methods

### Muscle Fiber Preparation.

Approximately 6-mm-long segments of demembranated fibers dissected from rabbit psoas muscle, a nearly pure fast-twitch muscle (33), were mounted in a custom-built fluorescence polarization setup between the lever arms of a force transducer and a loudspeaker motor in a temperature-controlled multidrop system (15). Fiber extremities were fixed using 5% glutaraldehyde in rigor solution and glued to the aluminum clips with shellac dissolved in ethanol. The initial sarcomere length was set at 2.45 µm, and the fiber cross-sectional area (CSA) was determined using a 40× wet objective. A total of 36 muscle fibers, with average CSA of 3,891 ± 154 µm^2^ (mean ± SE), isolated from five rabbits were used in these experiments.

### Protein Expression/Purification.

The E56C/E63C (C-helix) and E96C/R103C (E-helix) double-cysteine mutants of chicken skeletal TnC and the D95C/V103C (E-helix) double-cysteine mutant of chicken skeletal RLC of myosin were obtained by site-directed mutagenesis, expressed in *Escherichia coli*, and purified as previously described (17, 34). Each pair of introduced cysteines in the TnC and RLC mutants was cross-linked with bifunctional rhodamine [BR (35)] or bifunctional sulfo-rhodamine (BSR, B10621, Invitrogen), respectively, to give 1:1 BR:TnC and BSR:RLC conjugates that were purified to greater than 95% homogeneity. Specificity and stoichiometry of BR/BSR labeling were determined by mass spectrometry. Approximately 30% of the native TnC and RLC in the muscle fiber were replaced with TnC and RLC probes after incubation in EDTA-rigor buffer for 40′ at 19 °C containing ∼20 µM of labeled protein, as described previously (15, 17).

### Mechanical Protocols.

All the experiments were performed at 26 °C in the presence of 5% (w/v) Dextran T-500, conditions in which the physiological resting structure of the thick filament and the myofilament lattice spacing are recovered (17, 19). Active force was normalized by the CSA of each fiber in the absence of Dextran. The polarized fluorescence intensities were recorded first in relaxed fibers using X- and Y-illumination (λ = 532 nm) (15) to determine the order parameter <*P*_2d_> which quantifies the rapid probe motion (20). <*P*_2d_>= 0.931 ± 0.009 (mean ± SE, n = 6) for the TnC-E probe, 0.877 ± 0.008 (mean ± SE, n = 9) for the TnC-C probe, and 0.895 ± 0.007 (mean ± SE, n = 9) for the RLC-E probe, indicating that the amplitude of such motion is small.

In the calcium titration experiments, fibers exchanged with TnC or RLC probes were briefly (~1 to 5 s) activated by transfer from preactivating to activating solution at different pCa (=−log[Ca^2+^]) in the presence and in the absence of 25 µM blebbistatin, and the polarized fluorescence intensities obtained using X-illumination were used to calculate the second- and fourth-rank order parameters of the orientation distribution of the BR/BSR dipole, <*P*_2_> and <*P*_4_> respectively, and the total fluorescence intensity (15). Solutions with different pCa were obtained by mixing different proportions of activating (pCa 4.7) and relaxing (pCa 9.0) solutions, and the final pCa was calculated using software kindly provided by E. Homsher. The composition of preactivating, activating, and relaxing buffers has been described previously (17).

For experiments with caged calcium (NP-EGTA, N6802, Invitrogen), a UV pulse from a frequency-doubled ruby laser (λ = 347 nm, Lumonics Ltd, UK) circularly polarized with a λ/4 waveplate was attenuated via a quartz beamsplitter and microscope slide attenuators to ~60 mJ and vertically focused on the fiber with a cylindrical lens. The horizontal dimension of the beam was shaped with vertical slits to match the clip-to-clip length of each fiber. Before the photolysis, the fiber was incubated in preactivating solution for ~5 min to remove most of the EGTA from the relaxing solution and then transferred to the caged calcium solution for another 5 min before the UV flash. The caged calcium solution had the following composition: 25 mM imidazole, 5.55 mM Na_2_ATP, 20 mM sodium creatine phosphate, 6.58 mM MgCl_2_, 10 mM HDTA, 11 mM potassium propionate, 10 mM glutathione (reduced), and 1.8 mM NP-EGTA, pH 7.1, ionic strength = 150 mM. The calcium loaded in this solution from a 20 mM stock of CaCl_2_ was ∼1.5 mM to set pCa ∼ 6.8 and an active force of ∼2 to 3% of the maximum isometric force. The activation level produced by the flash was estimated by comparing the force and <*P*_2_> for the probe after photolysis with those obtained after transferring the fiber to activating solution 0.6 s after the flash. Only fibers which produced maximal activation were included in the analysis. The order parameters of the probe were sampled at 8 kHz (=0.125 ms per point). Fast photomultiplier tube modules (PMT H10723-20; Hamamatsu; frequency bandwidth DC-200kHz) were used to record the polarized intensities (sampling frequency 200 kHz). With these PMTs, the duration of the light artifact caused by the UV flash was ∼0.15 ms, about 10 times lower than that in a previous study using PMTs with slower response (15), increasing the time resolution of the fluorescence polarization measurement immediately after the UV photolysis. In the stretch-experiment, a stretch of amplitude ∼1% *L*_0_ complete in 0.3 ms was applied at 7 ms after the photolysis and was followed by an exponential stretch of amplitude ∼5% *L*_0_ with a time constant of ∼6 ms. The characteristics of the stretch were chosen to mimic the lengthening of the titin molecule in muscle fibers at constant force and at maximal calcium concentration in the presence of blebbistatin (see Fig. 3 in ref. 10).

### Data Analysis.

In the calcium titration experiments, the pCa values for TnC-C probes were offset by +0.1, corresponding to the shift in calcium sensitivity (pCa_50_) of force introduced by this probe, with respect to that measured in unexchanged fibers and in fibers exchanged with either the RLC or TnC E probe (Fig. 1*A*, black and magenta circles, respectively) at 26 °C in the presence of Dextran, much smaller than that observed previously at 11 °C in the absence of Dextran (16). The calcium dependence of force and <*P*_2_> was fitted with a single-Hill curve [y = B_pCa9_+A*(1/(1+(10^((x-pCa_50_)*n_H_))))]. The <*P*_2_> for TnC probes in the A-band of the sarcomere was calculated according to the equation <*P*_2_>_A-band_= (<*P*_2_>_obs_- (f_I-band_ ·<*P*_2_>_I-band_))/(1- f_I-band_), in which <*P*_2_>_obs_ is the observed <*P*_2_> in the absence of blebbistatin, f_I-band_ is the fraction of probes in the I-band, and <*P*_2_>_I-band_ is the <*P*_2_> for probes in the I-band and is assumed to be equal to the <*P*_2_> measured in the presence of blebbistatin. Considering that the length of the thin filament is 1.16 µm (36), the fraction of probes in the I-band (f_I-band_) at 2.45-µm sarcomere length was assumed to be 0.33, corresponding to the fractional length of the thin filament in the I-band. The probes in the I-band were assumed to be sensitive to only calcium, although it cannot be excluded that TnC probes near the A-I band junction might be partially sensitive to the myosin-induced structural changes in the A-band due to cooperativity of the myosin-dependent structural changes along the thin filament (Table 1). The calcium dependence of <*P*_2_>_A-band_ for the TnC-E probe was fitted with a double-Hill curve [y = B_pCa9_+ A_M_*(1/(1+(10^((x-pCa_50__M_)*n_H_M_))))+A_Ca_*(1/(1+(10^((x-pCa_50__Ca_)*n_H_Ca_))))], which included myosin (M)- and calcium (Ca)-dependent components (Table 1). The pCa_50_ and n_H_ for the calcium-dependent component (Ca) were constrained to the values determined in the presence of blebbistatin.

In the photolysis experiments, the UV flash produced a ~10% irreversible probe bleaching, associated with an instantaneous 10 to 15% change in <*P*_2_> toward 0, likely due to the rhodamine absorption band at ~350 nm close to the wavelength of the UV pulse. To compensate for this, the <*P*_2_> values before the flash were replaced by those measured in relaxing solution following photolysis. Isometric force decreased by ~15% after each UV flash. The time courses of <*P*_2_> for the TnC-C probe and TnC-E probe were fitted with three- and single-exponential functions, respectively, where *A_i_* and *r_i_* denote the amplitude and rate constant of the *i*-th exponential process (*SI Appendix*, Table S1).

### Statistical Analysis.

Error bars on mean data points are ± SEM. For mean data in the figures, the number of independent observations (n) corresponds to the number of muscle fibers used and is reported in the legends. The comparison of the goodness of the fit with single- and double-Hill curves was performed using a BIC test available in OriginPro (OriginLab Corp., Northampton, MA, USA). The BIC value for the double-Hill fit on the TnC-E probe data (−17.5) was much lower than that for the single-Hill fit (2.1), indicating that the double-Hill curve provides a better fit to the data (*SI Appendix*, Fig. S2). Paired Student’s *t* tests were used to determine the significance of differences in the parameters of the Hill fits (Table 1) and in the half-times of force and orientation changes of the probes (*SI Appendix*, Table S1).

## Supplementary Material

Appendix 01 (PDF)

## Data Availability

All relevant data, associated protocols, and materials are described within the manuscript and its *SI Appendix*. The code used for the mathematical model of the sarcomere simulating force and fraction of folded motors during contraction is available at https://github.com/lorenzomarcucci/FMM_model. The raw data from mechanical and fluorescence experiments are available at https://doi.org/10.5281/zenodo.7668420. All study data are included in the article and/or *SI Appendix*.
