# Supplementary material for: Activation of skeletal muscle is controlled by a dual-filament mechano-sensing mechanism
Source: Proc Natl Acad Sci U S A. 2023 May 22;120(22):e2302837120. doi: 10.1073/pnas.2302837120 (PMC10235942; doi:10.1073/pnas.2302837120)
Supplement: Supplementary file 1 — Appendix 01 (PDF) [file pnas.2302837120.sapp.pdf]

Supporting Information for

Activation of skeletal muscle is controlled by a dual-filament mechano-sensing mechanism.

Elisabetta Brunello, Lorenzo Marcucci, Malcolm Irving, Luca Fusi

corresponding author: Luca Fusi.
Email: luca.fusi@kcl.ac.uk

This PDF file includes:

Supporting text
Figures S1 to S6
Table S1
SI References

Supporting Information Text

Model simulation of the time course of force and of the OFF-to-ON transition of the myosin motors in a muscle fiber activated by photolysis of caged calcium.

The experimental time courses of force and of $<P_2>$ for the RLC-E probe, which is associated with the release of motors from the folded state during activation by photolysis of caged calcium were compared with the numerical simulations based on a Monte-Carlo type model of the myosin motors fluctuating in a thermal environment and interacting with the regulated actin filament, which includes the mechano-sensing activation of the thick filament (1). In this scheme, the myosin motors on the thick filament are in a tension-dependent equilibrium between folded/parallel (OFF) and perpendicular (ON) conformation with respect to the filament axis. In the perpendicular state, the myosin motors can enter the conventional cross-bridge cycle, attaching to the actin filament and performing a two-steps power stroke.

Firstly, we adapted the original model (1) to the muscle type and the temperature used in this study, by adjusting the rate constants of the conventional cross-bridge cycle to fit the rate of force generation after shortening (Fig.3 and Fig. S6), and to constrain the fraction of motors attached to actin at the isometric plateau to 0.3 (2). Then we implemented the original model using experimental constraints for the parameters of the mechano-sensing transition, by introducing a linear dependence on filament stress for the rate constant of the transition from the parallel to the perpendicular state (K_{ON}) similar to that observed for the activation of the myosin motors by passive tension in single muscle fibers (3), while the rate constant for the reverse reaction (K_{OFF}) was fixed to 45 s^{-1}. The value of K_{ON} at zero filament stress, K_{ON0}, was the only free parameter in the model and it was varied from 8 s^{-1} to 110 s^{-1} to adjust the fraction of constitutively ON motors in relaxing conditions from 15 to 70%. In each simulation the calcium concentration was initially increased from pCa 9 to pCa ~6.8 and a steady force of ~2% of the maximal isometric force, similar to the experimental value, and a steady fraction of constitutively ON motors were attained after an equilibration period of 1.5 s. At the time of the UV-photolysis (t=0 ms) the calcium concentration was increased to pCa 5.0 and the activation of the troponin-tropomyosin regulatory units on the thin filament was 90% complete in about 2 ms. The simulations were performed using 1800 pairs of thin-thick filaments to increase signal-to-noise ratio, linked to the component accounting for the thermal fluctuations in the model. The actin filament position was fixed during isometric contraction and was shifted towards the center of the sarcomere by 60 nm in 2 ms with constant velocity to simulate the experimental protocol. The active filament stress experienced by each myosin motors on the thick filament is calculated at each time step summing the actual force generated by the attached myosin motors from that point toward the M-band (4). The filament stress decreased almost linearly with the distance from the midpoint of the filament in the M-band, where the force is maximal, and was zero at its tips.

The simulations show that at low fractions of constitutively ON motors the time course of force generation after the calcium release (rising phase) is slower than that of force redevelopment after unloaded shortening, due to the kinetics of activation of the folded motors on the thick filament (Fig. S6). Increasing the fraction of constitutively ON motors reduced the lag in the rising phase of force and in the activation of the folded motors, and increased the speed of force generation after photolysis. The experimental time course of the force rise and of the early phase of activation of the folded motors estimated by the $<P_2>$ for the RLC-E probe could be simulated with a fraction of constitutively ON motors of ~35% (Fig. S6, green traces). However, at 20 ms after the photolysis, the simulated time course of the folded motors became slower than that of $<P_2>$, suggesting that the parameters of the mechano-sensing mechanism assumed in the model do not explain the kinetics of thick filament activation at higher loads. This result suggests that, while the filament stress triggers the activation of the myosin motors on the thick filament, an alternative mechanism, which might account for the high cooperativity of the activation of the myosin motors at steady calcium concentrations (Fig. 1), might contribute to the fast transmission of the activation signal for the motors along the thick filament. The partial recovery of the folded OFF state during shortening estimated by the model does not follow the change in $<P_2>$ for the

RLC-E probe induced by the shortening, due to the sensitivity of this probe to the myosin working stroke in the actin-attached myosin motors.

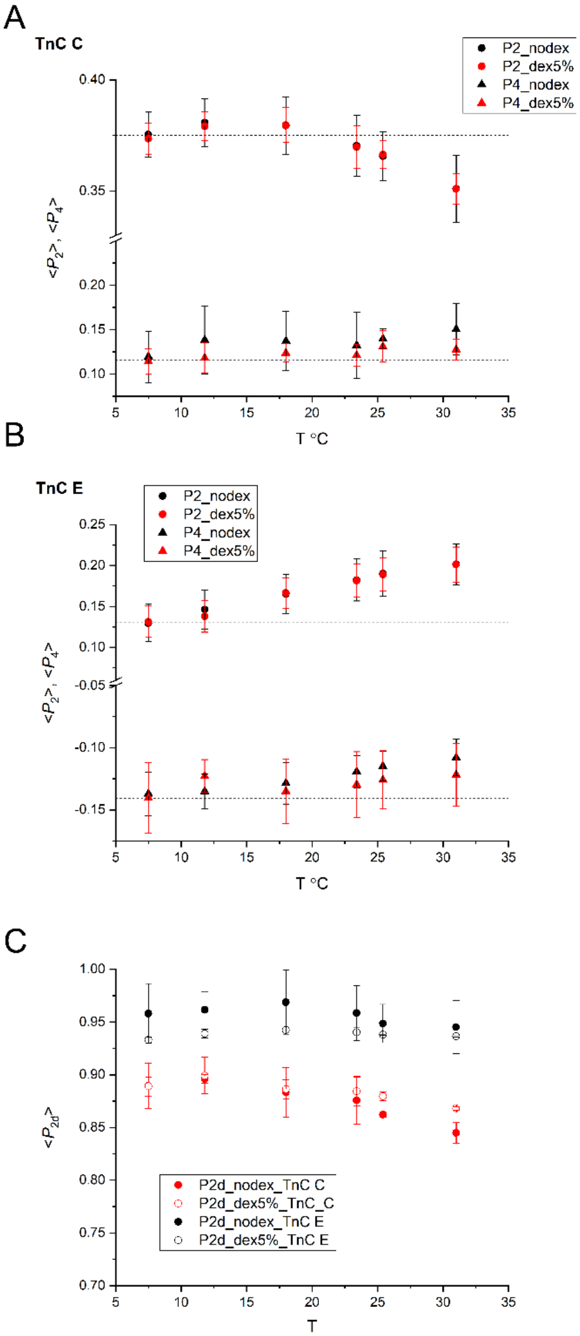

Fig. S1 Temperature dependence of TnC probe orientation in relaxed muscle fibers. Order parameters <P_2> (circles) and <P_4> (triangles) for TnC-C probe (A) and TnC-E probe (B) in the absence (black) and in the presence (red) of 5% (w/v) Dextran T-500 in relaxed muscle fibers (pCa 9.0). Horizontal dashed lines, mean values of the order parameters at 7°C. (C) Order parameter <P_{2d}> for TnC-C probe (red) and TnC-E probe (black) in the absence (filled symbols) and in the presence (empty symbols) of 5% (w/v) Dextran T-500. Data points are mean ± SD (n= 3 fibers).

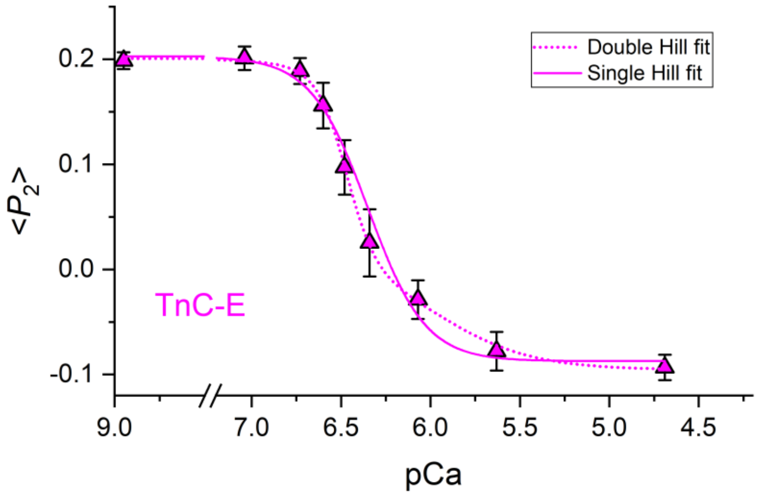

Fig. S2 Single- and double-Hill fits of the calcium dependence of <P_2> for TnC-E in the A-band. Order parameters <P_2> calculated for TnC-E probes in the A-band as in Fig.1D. Solid and dotted lines, single- and double-Hill curves fitted to the data, respectively (see Methods). The Bayesian information criterion (BIC) test indicates that the double-Hill curve (BIC value= -17.5) provides a better fit to the data than the single-Hill fit (BIC value=2.1).

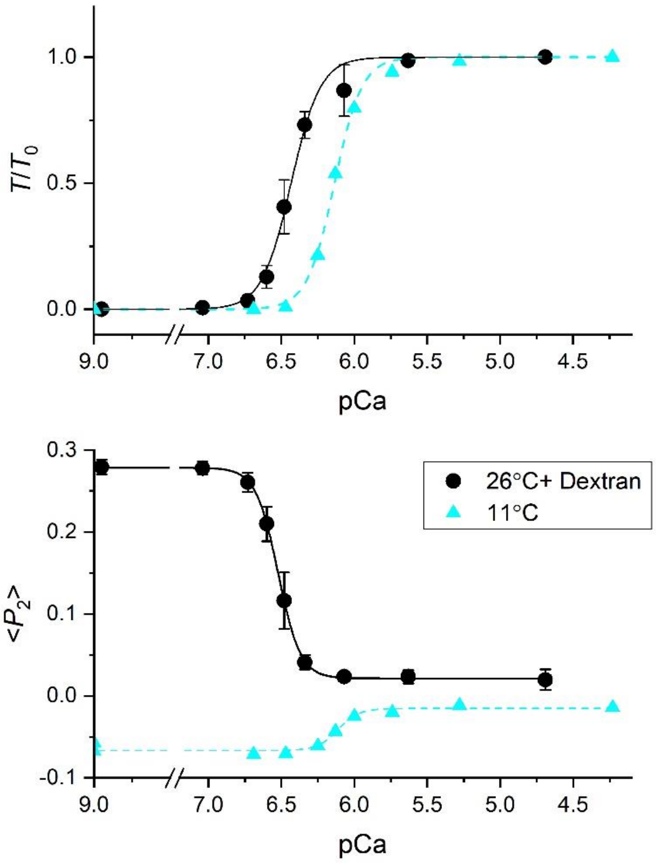

Fig. S3. Effect of cooling on the calcium dependence of force and RLC-E probe orientation. Relative force and order parameter <P_2> for the RLC-E probe at 26°C in the presence of 5% (w/v) Dextran T-500 (black circles, data from Fig. 1B) and at 11°C in the absence of Dextran (cyan triangles, n=1). T_0 is 243 and 168 kPa, at 26°C and 11°C respectively.

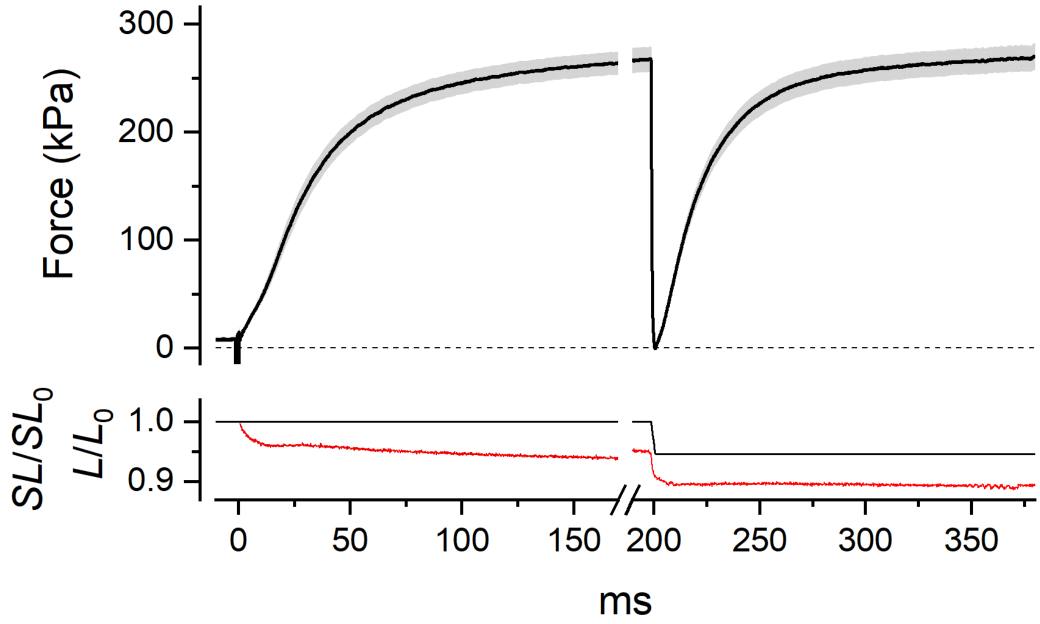

Fig. S4. Force and sarcomere length change in single muscle fibers during activation by photolysis of caged calcium and after ramp shortening. Time course of force (in kPa, mean trace ± SE in grey, n=14 fibers), fiber length (*L*, relative to the initial fiber length L_0; black) and sarcomere length (*SL*, relative to the initial sarcomere length SL_0 =2.45µm; red, mean trace, n=11 fibers) in muscle fibers activated by photolysis of caged calcium at time zero at 26°C in the presence of 5% (w/v) Dextran T-500. SL/SL_0 was calculated as the reciprocal of the relative fluorescence intensity *I* ($SL/SL_0 = I_0/I$), that is associated with the number of sarcomeres in the excitation volume (3). Horizontal dashed line marks the zero force.

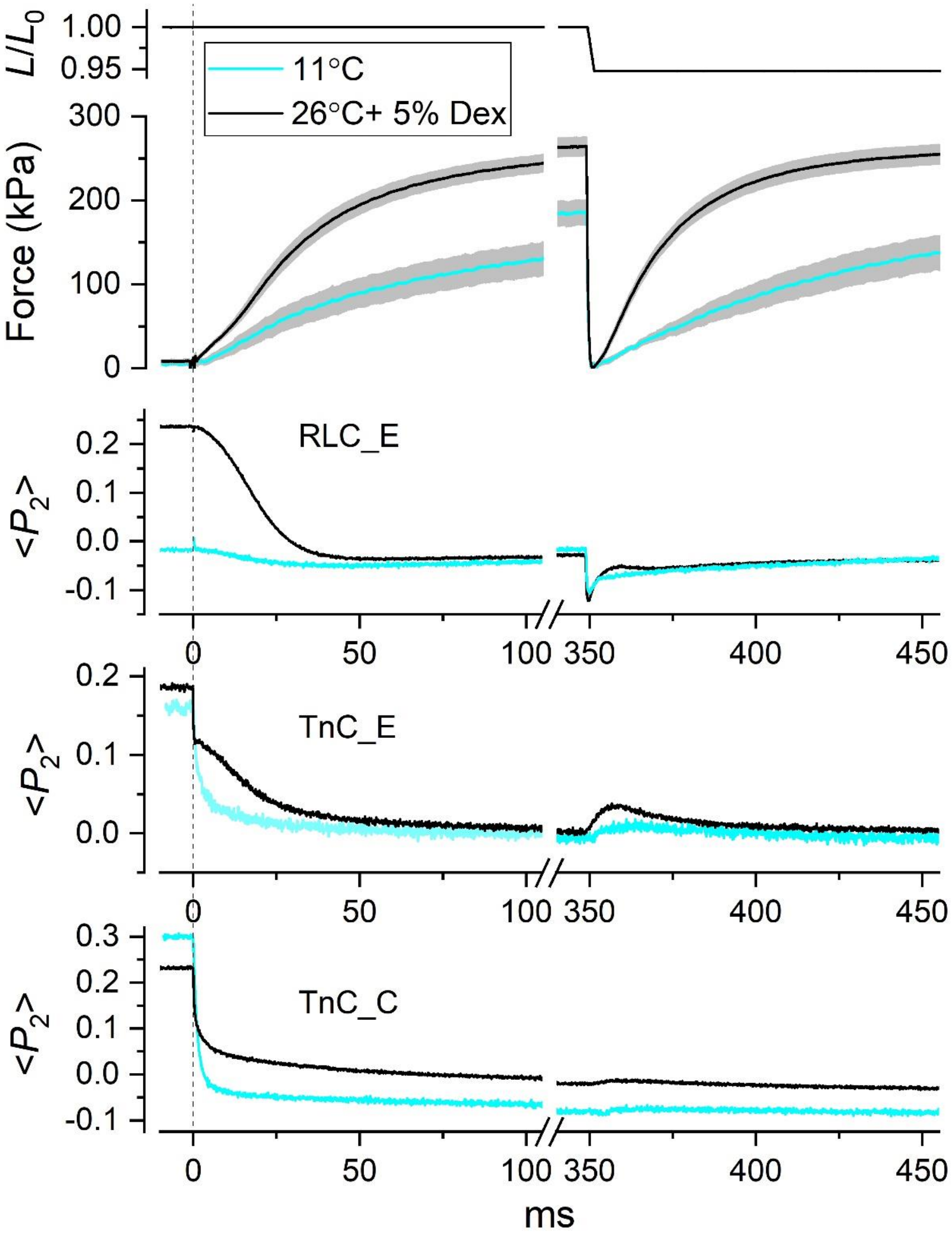

Fig. S5. Effect of cooling on the structural dynamics of TnC and RLC probes. Upper panel, fiber length *L* relative to the initial length L_0. Time courses of force in kPa and $<P_2>$ for RLC and TnC probes after the UV-photolysis of caged calcium at time zero (vertical dashed line), at 26°C in the presence of 5% (w/v) Dextran T-500 (black traces, data from Fig. 3) and at 11°C in the absence of Dextran (cyan traces; force, average ± SE in grey, and average $<P_2>$ for RLC-E probe, n=3 fibers; TnC data are from Fusi et al. (2014)(5)).

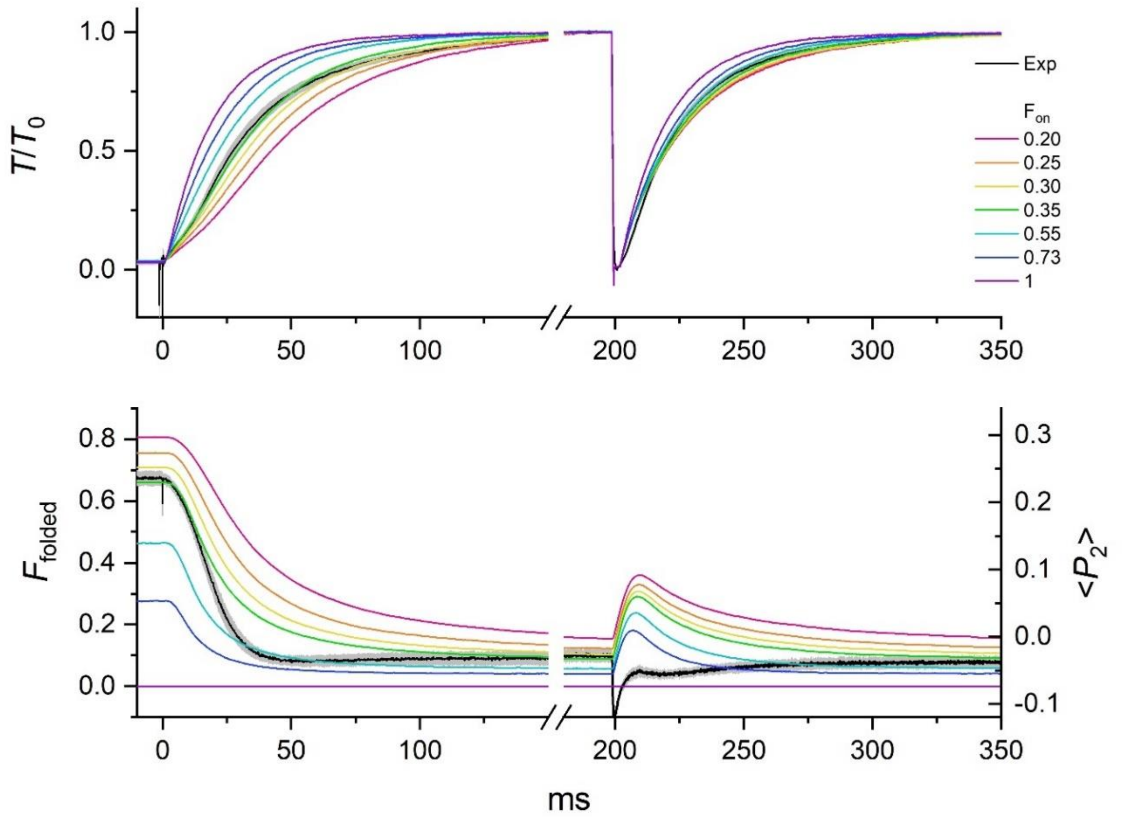

Fig. S6. Model simulation of the kinetics of force generation and of the OFF-to-ON transition of the myosin motors during contraction. Simulation of the time course of force (upper panel) and of the fraction of folded motors on the thick filament (F_{folded}; lower panel) during activation by photolysis of caged calcium at time zero and after ramp shortening at 200 ms, with different fractions of constitutively ON motors before activation (F_{on}=1- F_{folded}). Experimental time courses for force and <P_2> for the RLC-E probe from Fig. 3 are shown in black with SE in grey.

	t_{50} (ms)	i	r_i (s^{-1})	A_i
Force rise	29 ± 2	1	29.1 ± 2.1	1 (262±10 kPa)
Force redev	18 ± 1†	1	42.1 ± 1.6	1 (261±11 kPa)
< P_2 > TnC-C	-	1	3841 ± 504	0.40 ± 0.02
	-	2	371 ± 48	0.32 ± 0.04
	-	3	21 ± 2	0.28 ± 0.04
< P_2 > TnC-E_Ca	-	1	7100 ± 1280	0.36 ± 0.06
< P_2 > TnC-E_M	17 ± 2	-	-	-
< P_2 > RLC-E	18 ± 1†	-	-	-

Table S1. Time courses of force and of < P_2 > for TnC and RLC probes in muscle fibers activated by photolysis of caged calcium. Half-times (t_{50}; ms) for the time courses of force rise after photolysis of caged calcium and of force redevelopment after ramp shortening, and of < P_2 > for the RLC-E probe and for the myosin-dependent component of < P_2 > for TnC-E probe (< P_2 > TnC-E_M) at 26°C in the presence of 5% (w/v) Dextran T-500 (Fig. 3). < P_2 > for TnC-C probe was fitted with a three-exponential function. Force rise and redevelopment and the calcium-dependent component of < P_2 > for TnC-E probe (< P_2 > TnC-E_Ca) were fitted with single-exponential functions. Rate constants (r_i), relative amplitude (A_i) for each exponential component (i) were estimated from the fit. Mean ± SE, force n=15 fibers, < P_2 > TnC-C n=4 fibers, < P_2 > TnC-E n=4 fibers, < P_2 > RLC-E n= 7 fibers. † $P<0.05$ when comparing half-times of force redevelopment and < P_2 > with that of force rise using a t-test.

SI References

1. L. Marcucci, C. Reggiani, Mechanosensing in Myosin Filament Solves a 60 Years Old Conflict in Skeletal Muscle Modeling between High Power Output and Slow Rise in Tension. *Front Physiol* **7**, 427 (2016).
2. M. Linari, M. Caremani, C. Piperio, P. Brandt, V. Lombardi, Stiffness and fraction of Myosin motors responsible for active force in permeabilized muscle fibers from rabbit psoas. *Biophys. J.* **92**, 2476-2490 (2007).
3. L. Fusi, E. Brunello, Z. Yan, M. Irving, Thick filament mechano-sensing is a calcium-independent regulatory mechanism in skeletal muscle. *Nat Commun* **7**, 13281 (2016).
4. L. Marcucci, T. Washio, T. Yanagida, Proposed mechanism for the length dependence of the force developed in maximally activated muscles. *Sci Rep* **9**, 1317 (2019).
5. L. Fusi, E. Brunello, I. R. Sevrieva, Y. B. Sun, M. Irving, Structural dynamics of troponin during activation of skeletal muscle. *Proc Natl Acad Sci USA* **111**, 4626-4631 (2014).
